# *Gracilaria gracilis* Aqueous Extract and White *Chlorella* as Functional Ingredients in Yogurt: Effects on Quality, Nutritional and Physicochemical Properties

**DOI:** 10.3390/foods15193405

**Published:** 2026-09-24

**Authors:** Gabriela Kovaleski, Andreia S. Ferreira, Cláudia Nunes, Sónia P. M. Ventura

**Affiliations:** 1 Department of Chemistry, CICECO—Aveiro Institute of Materials, University of Aveiro, Campus Universitário de Santiago, 3810-193 Aveiro, Portugal; gabrielak@ua.pt; 2 Department of Chemistry, LAQV-REQUIMTE, University of Aveiro, Campus Universitário de Santiago, 3810-193 Aveiro, Portugal; a39493@ua.pt; 3 Department of Materials and Ceramic Engineering, CICECO—Aveiro Institute of Materials, University of Aveiro, Campus Universitário de Santiago, 3810-193 Aveiro, Portugal; claudianunes@ua.pt

**Keywords:** yogurt, nutritional value, natural coloration, firmness, phenolic content, antioxidant activity

## Abstract

The growing demand for functional foods has increased interest in natural ingredients with nutritional and bioactive properties for dairy applications. This study evaluated *Gracilaria gracilis* (*G. gracilis*) aqueous extract and White *Chlorella* as functional ingredients in yogurt. Three formulations were prepared: control yogurt (CY), yogurt containing *G. gracilis* extract (GY), and yogurt containing both *G. gracilis* extract and White *Chlorella* (GWY). Proximate composition, total phenolic content (TPC), antioxidant activity, color, pH, texture, and water-holding capacity (WHC) were assessed after fermentation and over 21 days of refrigerated storage. GY exhibited a pink coloration that was clearly perceptible relative to the control (ΔE* > 3) and remained stable throughout storage (ΔE* < 0.6 relative to Day 1). GY also showed lower firmness, consistency, cohesiveness, and WHC than CY, differences that may be attributable, at least in part, to the dilution introduced with the aqueous extract. Compared with GY, GWY contained 32% more protein (3.66 vs. 2.78 g/100 g) and showed higher firmness, consistency, and WHC, in some cases reaching values comparable to those of the control. TPC did not differ significantly among formulations (*p* > 0.05), while the antioxidant activity of both supplemented yogurts was lower than that of the control. Instrumental color changes during storage were small in magnitude, while texture remained generally stable across the 21-day storage period.

## 1. Introduction

In recent years, the demand for functional and naturally fortified foods has increased significantly, driven by consumer preference for healthier and more sustainable products [1,2]. In this context, the development of novel formulations based on natural ingredients has emerged as a promising strategy to reduce or replace synthetic additives, minimize environmental impact, and enhance the nutritional and sensory quality of food products. This trend is aligned with the increasing consumer awareness of clean-label and health-conscious foods [3,4]. However, identifying ingredients that provide both functional and sensory benefits without compromising stability remains a key challenge.

Algae, including both micro and macroalgae, have gained significant attention as nutrient-rich and sustainable food ingredients. These photosynthetic organisms, whether prokaryotic or eukaryotic, thrive in saline or freshwater environments and are rich sources of proteins, polysaccharides, lipids, vitamins, minerals, and pigments [5,6]. Among macroalgae, *Gracilaria gracilis*—*G. gracilis* (Rhodophyta) has emerged as an economically important resource due to its high content of agar and phycobiliproteins, particularly phycoerythrin. This pigment is known for its pink color and potential use as a natural food dye [7,8]. However, limitations related to the thermal and photostability of these natural pigments could restrict their application in food systems [9]. Microalgae, such as *Chlorella vulgaris*, are also widely recognized for their high nutritional value, particularly their protein content, which can exceed 50% of dry weight, as well as their vitamin and antioxidant composition [10,11]. Nevertheless, their intense green color and characteristic flavor can negatively affect the sensory properties of food products when incorporated at higher concentrations [12]. Recently, White *Chlorella*, a chlorophyll-deficient form of *C. vulgaris*, has emerged as an alternative food ingredient characterized by a reduced pigment content, resulting in a lighter coloration and a milder sensory profile compared with conventional green *C. vulgaris*, potentially broadening its applicability in food formulations [13]. *C. vulgaris* has a documented history of significant consumption as food in the European Union prior to 15 May 1997 and is therefore considered a non-novel food, not requiring pre-market authorization under Regulation (EU) 2015/2283 [14,15]. *Chlorella* biomass is well established in the European health-food and dietary-supplement market and is commonly marketed as a nutrient-rich food ingredient. White *C. vulgaris* biomass is already commercially available as a food-grade product marketed for human consumption as a food supplement in Europe (https://www.allmashop.com/en/produto/clorela-branca-em-po-100g-25un/?srsltid=AfmBOoqeliLTqrmrUC9bS5jF9TXiGpoOwaMmMc1aza2ZOOZhqvDk5BRE) accessed on 1 August 2026.

Despite the growing interest in algae-based food ingredients, most studies have focused on the direct incorporation of the whole biomass, whereas the use of algal extracts remains underexplored [16,17]. In the case of pigmented macroalgae, whole biomass carries the color but also the fibrous texture, characteristic flavor and dark particles that limit consumer acceptance, whereas most reported phycobiliprotein extracts are obtained through complex protocols involving lyophilization, cell disruption, ammonium sulfate precipitation and dialysis, which are costly and difficult to scale [8,18]. A crude aqueous extract represents an intermediate approach: extraction with water alone, at room temperature and in a single short step, avoids organic solvents and energy-intensive downstream processing and yields a liquid extract that can be incorporated directly into a dairy matrix. The trade-off is a less purified extract, which was considered acceptable here because the intended function is coloring rather than delivery of an isolated pigment. Studies combining macro- and microalgae within the same food matrix remain scarce. Pehlivan et al. [19] incorporated *C. vulgaris* together with the red macroalga *Chondrus crispus* into ayran, a traditional fermented milk beverage, and reported a significant increase in protein content that was not observed when the microalga was added alone, suggesting that the two types of biomasses may contribute with complementary nutritional and technological properties. To the best of our knowledge, however, the combination of a macroalgal aqueous extract used as a natural coloring agent with a microalgal biomass used as a protein source has not previously been reported in yogurt. This combination is of particular interest because both ingredients provide distinct formulation characteristics: the aqueous extract contributes with coloration without incorporating the whole macroalgal biomass, whereas White *Chlorella* provides a protein-rich biomass with a markedly lower pigment content than conventional green *C. vulgaris*.

Yogurt represents an appropriate matrix for the incorporation of functional ingredients because of its nutritional value, widespread consumption, and suitability for fortification. However, the incorporation of algal ingredients may affect fermentation, texture, color, and product stability and therefore requires careful evaluation. Accordingly, the aim of this study was to develop and characterize yogurt formulations containing *G. gracilis* aqueous extract, either alone (GY) or together with White *Chlorella* (GWY), and to compare their nutritional, physicochemical, and functional properties with those of a control yogurt (CY). Particular attention was given to the color stability of the *G. gracilis*-containing formulations and to the compositional and physicochemical differences between GY and GWY during refrigerated storage. It was hypothesized that the aqueous *G. gracilis* extract would provide a stable natural coloration and that the formulation additionally containing White *Chlorella* would exhibit a higher protein content than GY while allowing its physicochemical behavior during storage to be evaluated. This formulation-based approach was intended to assess the technical feasibility of incorporating algae-derived ingredients into a yogurt matrix and to characterize the resulting formulations instrumentally.

## 2. Materials and Methods

### 2.1. Materials

Fresh *G. gracilis* was supplied by ALGAplus Lda, an aquaculture company located in Ílhavo, Portugal. White *Chlorella* is a chlorophyll-deficient strain of *Chlorella vulgaris* Beijerinck, supplied as a gently spray-dried powder by Allmicroalgae—Natural Products S.A. (Pataias, Portugal), under the commercial designation “White *Chlorella vulgaris*” (batch nº. L23WC019). This strain was derived from a heterotrophic *C. vulgaris* strain through chemically induced random mutagenesis [20] and is produced by heterotrophic fed-batch fermentation [21]. The resulting reduction in pigment content contributes to its lighter color and milder sensory profile compared with conventional autotrophic *C. vulgaris* [20,22]. The biomass used in the present study is commercially marketed for human consumption as a food supplement.

For this alga, carbohydrate, protein, moisture and ash analyses were performed. UHT whole milk (Terra Nostra^®^, Azores, Portugal) was purchased at Auchan, Aveiro, Portugal. The commercial yogurt used for comparison of the pink color was Clesa^®^ strawberry flavor (Cooperativas Lácteas Unidas, Galicia, Spain) (containing E-120 dye and E-100; nutritional values per 100 g: energy: 334.00 kJ (80.00 kcal); lipids: 2.00 g, of which saturated fatty acids: 1.30 g; carbohydrates: 12.50 g, of which sugars: 12.50 g; proteins: 3.00 g; salt: 0.13 g; calcium: 120.00 mg).

Glucose (99.5% of purity, CAS G8270), gallic acid (98.0% of purity, CAS 398225), methanol (99.8% of purity, CAS 179337), 3-ethylbenzothiazoline-6-sulfonic acid (98% of purity, CAS A1888), potassium persulfate (98% of purity, CAS 216224) and Whatman filter paper Nº 1 (110 mm) were purchased at Sigma-Aldrich (St. Louis, MO, USA).

### 2.2. Pre-Treatment of G. gracilis Biomass and Aqueous Extraction

The aqueous extraction workflow was adapted from that previously used for phycobiliprotein recovery from *Gracilaria* biomass supplied by the same producer [23], with the solid-to-liquid ratio adjusted in the present study to obtain a concentrated extract while minimizing the volume of water subsequently introduced into the dairy matrix. The batch of fresh *G. gracilis* used in this study was produced in January 2024. After collection, the macroalgae samples were washed and stored in a freezer at −20 °C. The frozen biomass was macerated in a mortar and pestle with liquid nitrogen. For each extraction, 20 g of ground fresh biomass was mixed with 40 mL of distilled water at room temperature, corresponding to a solid–liquid ratio of 1:2 (*w*/*v*, fresh-weight basis). This ratio was selected as the lowest allowing complete wetting and homogeneous stirring of the ground biomass, thereby enabling the recovery of a concentrated extract while minimizing the volume of water subsequently introduced into the dairy matrix. The mixture was placed in an incubator shaker (IKA KS 4000 ic control) (IKA, Staufen, Germany) for 20 min at 250 rpm. Subsequently, the mixture was filtered and centrifuged (Thermo Scientific Heraeus Megafuge 16 R Centrifuge) (Thermo Fisher, Osterode am Harz, Germany) at 3500 rpm for 30 min at 4 °C, after which the resulting crude liquid extract was recovered. On average, 33 mL of crude liquid extract was recovered per extraction, corresponding to 82.5% of the water added. The extract was used immediately after preparation. The dry matter content of the extract was 1.66 g/100 mL, giving an extraction yield of 2.74 g of dry extract per 100 g of fresh biomass. Since the moisture content of the fresh biomass was not determined, the yield is expressed on a fresh-weight basis. For the aqueous extract, carbohydrate, protein, moisture, ash, and total phenolic compound contents, as well as antioxidant activity, were determined (Table 1).

### 2.3. Yogurt Preparation

The yogurts were prepared using 0.3 L of UHT whole milk (Terra Nostra^®^, Azores, Portugal) combined with 125 g of commercial natural yogurt (Alcampo, Auchan Retail España, S.L., Madrid, Spain. Nutritional values per 100 g: energy: 246 kJ (59 kcal); fats: 2.6 g, of which saturated: 1.7 g; carbohydrates: 4.9 g, of which sugars: 4.9 g; proteins: 3.9 g; salt: 0.14 g; starter cultures: *Streptococcus thermophilus* and *Lactobacillus delbrueckii subsp. bulgaricus*). The mixture was homogenized and heated to 43 °C for 3 min, and then the algae were added to this mixture. Two types of yogurts were produced: one with 5% (*v*/*v*) of *G. gracilis* aqueous extract (GY), and the other containing both 5% (*v*/*v*) of *G. gracilis* aqueous extract and 2% (*w*/*v*) of White *Chlorella* (GWY). Both percentages are expressed relative to the total volume of the milk–yogurt base prior to addition. The yogurt was then fermented in an oven (IKA) at 43 °C until the pH stabilized at approximately 4.3 (10–12-h). After fermentation, the yogurt was gradually cooled and stored at 6 °C until analysis. A control yogurt (CY), without algae addition, was also prepared for comparison purposes. Three independent production batches were prepared for each formulation (*n* = 3).

A commercial yogurt was used as inoculum, following a back-slopping procedure, rather than a standardized freeze-dried starter culture, since this approach reproduces the artisanal practice that the formulations were intended to emulate and has been shown not to compromise the fermentation process. Although the ratio selected is considerably higher than that typically employed with standardized starter cultures, it was established in preliminary trials: 125 g per 0.3 L of milk produced a markedly firmer set gel than lower inoculum proportions and was therefore chosen. A firm initial structure was considered advantageous given that the subsequent addition of a liquid extract was expected to weaken the gel network.

The incorporation levels were established in preliminary trials. For the *G. gracilis* aqueous extract, concentrations of 1, 5 and 10% (*v*/*v*) were tested, and 5% (*v*/*v*) was selected as the lowest concentration that produced a pink coloration instrumentally comparable to that of a commercial strawberry-flavored yogurt (ΔE* < 3.5; Table S2), thereby delivering the target color while minimizing the volume of water introduced into the dairy matrix. For White *Chlorella*, 2% (*w*/*v*) was selected as the upper limit reported in the literature for microalgal incorporation in fermented dairy products without adverse effects on consumer acceptance, since concentrations above this level have been associated with reduced acceptability of the final product [24,25].

#### 2.3.1. Proximate Analyses

Protein content was quantified by determining nitrogen through elemental analysis (LECO TruSpec Micro CHNS) (LECO Corporation, St. Joseph, MI, USA), with previously freeze-dried samples, using a nitrogen-to-protein conversion factor of 6.25 for the algae and 6.38 for the yogurt [26]. For moisture quantification, samples were dried in an oven at 102 °C until a constant mass was achieved. Ash content was measured as a percentage after incineration in a muffle furnace at 550 °C for 5 h (AOAC 1995). Carbohydrates were determined by the Dubois method [27] using glucose as a standard for the calibration curve (Figure S1 of the Supporting Information). These analyses were performed for the algae biomass, aqueous extract, and yogurt samples.

#### 2.3.2. Physico-Chemical Analysis

##### Color Evaluation

The color of yogurt samples was evaluated using a colorimeter (Tecnal) using illuminant D65 and UV WinLab software (Softmax Pro 7.2). Color measurements were expressed in the CIELAB color space using three values: L* (luminosity, where 0 represents black and 100 represents white), a* (the red/green coordinate, with +a indicating red and -a indicating green), and b* (the yellow/blue coordinate, with +b indicating yellow and -b indicating blue). The color difference (ΔE) was calculated according to Equation (1).

(1) $$\Delta E=\sqrt{{\left(L-L_{0}\right)}^{2}+{\left(a-a_{0}\right)}^{2}{+(b-b_{0})}^{2}},$$

where L*_0_, a*_0_, and b*_0_ represent the values for the CY, while L*, a*, and b* denote the measured values of each sample (GY and GWY) after the extract’s incorporation.

##### Water-Holding Capacity (WHC)

The WHC of the yogurts was measured by centrifuging 5 g of each sample at 4200 *g* for 20 min at 4 °C (Thermo Scientific Heraeus Megafuge 16R centrifuge). The WHC was calculated through Equation (2) [28]:

(2) $$WHC\left(\%\right)=\left(1-\frac{W1}{W2}\right)\times 100,$$

where W_1_ is the mass of the supernatant (whey) recovered after centrifugation (g) and W_2_ is the mass of the yogurt sample before centrifugation (g). Analyses were performed on one sample from each of the three independent production batches (*n* = 3).

#### 2.3.3. pH

The pH of the yogurts was measured after calibrating the pH meter (Metrohm, Herisau, Switzerland) with standardized buffer solutions at pH 4.0, 7.0, and 10.0 prior to analysis.

#### 2.3.4. Mechanical Properties

All instrumental texture analyses were performed on the yogurt samples at a temperature of 6 °C using a texture analyzer (Stable Micro Systems) (Stable Micro Systems Ltd., Godalming, UK). The tests were conducted directly in 50 g plastic sample cups, employing a 30 mm back extrusion probe. The samples were compressed to 70% of their original height, with a test speed of 0.50 mm·s^−1^ and a post-test speed of 10 mm·s^−1^. Hardness, consistency, cohesiveness, and work of cohesion values were calculated using the software provided by Stable Micro Systems. Texture measurements were performed on one sample from each of the three independent production batches (*n* = 3) at Days 1, 7, 14, and 21 of refrigerated storage.

#### 2.3.5. Determination of Total Phenolic Content (TPC)

Total polyphenols were extracted following the method described by Arfaoui 2020 [29]. Five grams of the yogurt sample was mixed with 45 mL of a methanol–water solution (50/50) and left to stir gently at room temperature for 2 h. The mixture was then filtered using a Whatman filter paper No. 1 and centrifuged at 4200 *g* for 30 min. The supernatant was collected and the TPC was determined using the Folin–Ciocalteu method [30]. The results were expressed as milligrams of gallic acid equivalent (GAE) per 100 g of sample, based on a calibration curve of gallic acid (Figure S2 in Supporting Information). For the yogurt samples, TPC was determined once in each of the three independent production batches (*n* = 3).

#### 2.3.6. Antioxidant Activity

The antioxidant activity of the yogurts was determined by the ABTS radical scavenging method [31]. The extraction of the samples was performed as previously described in Section 2.3.5. The radical cation was prepared by mixing 7 mM of 3-ethylbenzothiazoline-6-sulfonic acid (ABTS) with 2.45 mM of potassium persulfate and allowing the mixture to stand in the dark for 16 h. The resulting ABTS•^+^ solution was diluted with water to achieve an absorbance of 0.70 ± 0.05 at 734 nm. Subsequently, 0.05 mL of the sample was combined with 0.250 mL of ABTS•^+^, shaken for 5 min, and then the absorbance was measured at 734 nm. The ABTS radical scavenging inhibition percentage was calculated using Equation (3).

(3) $$I\left(\%\right)=\left[\frac{\left(A0-A1\right)}{A0}\right]\times 100,$$

where *A*0 is the absorbance of the control, and *A*1 represents the absorbance of the sample. For the yogurt samples, antioxidant activity was determined once in each of the three independent production batches (*n* = 3).

### 2.4. Statistical Analysis

All analyses were based on three independent production batches per formulation (*n* = 3), with one analytical determination performed per batch. For color measurements, four technical readings were obtained within each batch and averaged before statistical analysis. Technical replicates were therefore not treated as independent observations. Results are expressed as the mean ± standard deviation of the three independent production batches. Parameters assessed at a single time point—proximate composition, total phenolic content, and antioxidant activity were analyzed by one-way analysis of variance (ANOVA), with formulation as the fixed factor. Parameters monitored repeatedly during refrigerated storage, namely color coordinates and total color difference, pH, texture parameters, and water-holding capacity, were analyzed by two-way repeated-measures ANOVA, with formulation as the between-batch factor and storage time (Days 1, 7, 14, and 21) as the within-batch factor, including the formulation × storage time interaction. For pH, the pre-fermentation (BF) measurements were not included in the refrigerated-storage analysis and were analyzed separately by one-way ANOVA to compare formulations before fermentation. When significant effects were detected, pairwise comparisons were performed using Tukey’s honestly significant difference (HSD) test at *p* < 0.05, using the appropriate error term from the corresponding model. When a significant formulation × time interaction was observed, simple effects were examined. Comparisons among sampling or storage times within each formulation are indicated by lowercase letters, whereas comparisons among formulations at the same sampling or storage time are indicated by uppercase letters.

Model assumptions were evaluated using both formal tests and graphical diagnostics. Normality of model residuals was assessed using the Shapiro–Wilk test and inspection of normal probability plots, homogeneity of variances using Levene’s test, and sphericity of the within-batch factor using Mauchly’s test. The corresponding diagnostic results are provided in the Supporting Information.

Levene’s test indicated heterogeneity of variances for the L* coordinate and for firmness, consistency, and work of cohesion, whereas the residuals for a* showed a departure from normality. These deviations were mainly associated with the markedly different variability among formulations and with the greater between-batch variability observed for the control yogurt at Day 1. Given the fully balanced experimental design, ANOVA was considered sufficiently robust to deviations of this magnitude [32]. Nevertheless, because only three independent batches were available per formulation, formal diagnostic tests have inherently limited statistical power. Graphical inspection of model residuals was therefore used as a complementary assessment of model adequacy [33]. Complete ANOVA outputs, including F statistics, degrees of freedom, *p*-values, and effect sizes for the main effects and interactions, are provided in the Supporting Information. All statistical analyses were performed using Statistica 7 (StatSoft Inc., Tulsa, OK, USA).

## 3. Results and Discussion

Three yogurt formulations were produced to assess the incorporation of *G. gracilis* aqueous extract and White *Chlorella* into a fermented dairy matrix, namely a control yogurt (CY), a formulation containing *G. gracilis* aqueous extract (GY), and a formulation containing both *G. gracilis* aqueous extract and White *Chlorella* (GWY). The *G. gracilis* aqueous extract was obtained through biomass maceration followed by a solid–liquid extraction using water, thereby avoiding the use of organic solvents or additional costly downstream processing steps such as freeze-drying, while preserving its natural color. The concentration of *G. gracilis* aqueous extract was established at 5% (*v*/*v*), based on preliminary colorimetric comparison with a commercial strawberry-flavored yogurt. Furthermore, the microalga White *Chlorella*, available as a commercial product, was directly incorporated into the formulation without further processing. A concentration of 2% (*w*/*v*) of White *Chlorella* was chosen for incorporation into the yogurt formulation, since previous studies showed that higher concentrations have been associated with reduced consumer acceptance [24,25].

Both ingredients, macroalgae aqueous extract and microalga biomass, were characterized prior to incorporation. Their proximate composition (protein, carbohydrate, moisture, and ash), TPC, and antioxidant activity (ABTS assay) were determined. The results are summarized in Table 1.

The *G. gracilis* aqueous extract’s main component is carbohydrates, 0.28 g/100 mL on a wet basis, followed by protein at 0.22 g/100 mL. White *Chlorella* biomass exhibited a substantially high protein content (45.42 g/100 g, dry basis), confirming its potential as a protein-enriching ingredient. In addition, the microalgae showed a higher ash content (6.0 g/100 g), indicating a great mineral contribution. White *Chlorella* biomass contained also a considerable content of carbohydrates, 13.40 g/100 g.

The total phenolic content (TPC) was only determined in the *G. gracilis* aqueous extract, reaching 24.02 mg GAE/100 g (wet basis), which was associated with a higher antioxidant activity (17.47% inhibition, ABTS assay) compared to White *Chlorella* biomass (10.05%).

These results highlight the complementary functional roles of both ingredients: White *Chlorella* as a concentrated source of protein and micronutrients, and *G. gracilis* extract as a source of bioactive compounds with antioxidant potential, supporting their combined application in fortified yogurt formulations.

### 3.1. Proximate Analyses of Yogurt

The nutritional composition of the three yogurt formulations was evaluated in terms of carbohydrate, protein, moisture, and ash content (Table 2). GY and GWY showed lower carbohydrate and ash contents than CY, differences that are likely influenced by the dilution associated with incorporation of the liquid *G. gracilis* extract. Protein content did not differ significantly between CY and GY. In contrast, GWY contained significantly more protein than GY (3.66 vs. 2.78 g/100 g), corresponding to a 32% difference. Since GY and GWY contained the same concentration of *G. gracilis* aqueous extract and differed by the inclusion of White *Chlorella*, the higher protein content of GWY is consistent with the high protein content of the White *Chlorella* biomass (45.42 g/100 g, dry basis; Table 1). However, in the absence of a formulation containing White *Chlorella* alone, this comparison does not establish an independent effect of the microalgal biomass outside the *G. gracilis*-containing matrix.

To date, no studies have reported the incorporation of White *Chlorella* into dairy products, with the available literature focusing predominantly on conventional *Chlorella vulgaris* strains. Previous studies reported increased protein content following the incorporation of *C. vulgaris* into processed cheese at inclusion levels of 3 and 4% [25,34], whereas incorporation at 0.25% into ayran did not significantly alter protein content [19]. Pehlivan et al. [19] further reported a protein increase when *C. vulgaris* was combined with the macroalga *Chondrus crispus*, which the authors discussed in the context of possible complementary effects. The present experimental design, however, does not allow such an interaction to be tested. In the current study, GWY exhibited a significantly higher protein content than GY, consistent with the protein-rich composition of the White *Chlorella* biomass. These results demonstrate that the formulation containing both algal ingredients achieved a higher protein content than the formulation containing *G. gracilis* extract alone, without implying an independent or synergistic effect of White *Chlorella*.

### 3.2. Color Stability of Yogurt

The aqueous extract of *G. gracilis* was previously tested for color stability during lactic acid fermentation over a 12-h period (Table S1 in the Supporting Information). The total color difference relative to the blank, that is, the milk–yogurt base prepared without added extract, ranged from 7.33 to 8.17 and did not vary significantly throughout the 12 h fermentation period. This indicates that the color conferred by the *G. gracilis* extract was established immediately upon incorporation and remained relatively stable throughout acidification and gel formation. The pink coloration observed in GY is most plausibly associated with R-phycoerythrin. In a previous proteomic characterization of an aqueous crude extract prepared from *Gracilaria* biomass cultivated by the same producer (ALGAplus, Ílhavo, Portugal) using a closely comparable extraction workflow, R-phycoerythrin was identified as the predominant phycobiliprotein, with its α and β subunits accounting for 44.2% and 15.2%, respectively, of the relative abundance of the proteins identified in the crude extract [23]. R-phycocyanin and allophycocyanin were present at substantially lower relative abundances. Considering the common biomass source, cultivation system, and similar aqueous extraction procedure, R-phycoerythrin is therefore the most plausible major contributor to the pink coloration observed in the present yogurt formulation. A comparative assessment with a commercial strawberry flavor yogurt containing two colorants, E-100 (Curcumin) and E-120 (carmine), was performed (Table S2 in the Supporting Information). The GY sample exhibited a slightly more intense color, with a color difference (ΔE) of 2.88 ± 1.25, a value close to the threshold of 3 above which color differences become clearly perceptible to an untrained observer [34,35]. These results show that the instrumental color coordinates of GY were close to those of the commercial strawberry-flavored yogurt, thus demonstrating the suitability of *G. gracilis* extract as a natural coloring agent in fermented dairy systems. Based on this preliminary validation, the color stability of GY and GWY samples was monitored over 21 days of refrigerated storage (Table 3). Two-way ANOVA revealed a significant formulation × storage time interaction for all color parameters (*p* < 0.001; Table S4), indicating that both formulations followed different color trajectories during storage.

The GY sample retained the pink coloration imparted by the *G. gracilis* aqueous extract throughout storage. A small but significant decrease in a* was observed, from 3.87 at day 1 to 3.55 at day 21 (a reduction of 8%), whereas L* and b* did not change significantly (*p* > 0.05). The magnitude of this change was negligible in visual terms: the total color difference relative to day 1 of the same batch did not exceed 0.51 at any sampling point, well below the threshold of perceptibility for a trained observer. Relative to the control yogurt, ΔE* ranged from 5.71 to 6.02 (Table S3), consistently above the value of 3 at which color differences become clearly perceptible to an untrained observer, confirming that the pink coloration was both visually evident and stable.

In a related study, B-phycoerythrin extract from the microalga *Porphyridium cruentum* was incorporated into milk-based products, and its color stability was assessed [10]. Over an 11-day period, the color of the milkshake, liquid yogurt, and (solid) yogurt remained stable with no significant changes. However, the extraction protocol involved a multi-step process including lyophilization, buffer reconstitution, cell wall disruption, ammonium sulfate precipitation, dialysis, and the addition of sodium azide as a preservative. In contrast, our study used a crude aqueous extract of *G. gracilis*, avoiding purification steps and organic solvents. This simplified approach reduces processing time, cost, and waste generation, although it results in a less purified extract. Nevertheless, the higher incorporation level of the crude extract proved effective in maintaining color stability throughout storage, indicating technical potential for its use as a natural colorant [36].

The color parameters of the GWY sample (Table 3) revealed differences compared to the GY sample, with a decrease in a* value and an increase in b* values, indicating a shift toward a greener/yellower hue. This change can be attributed to the residual pigmentation of White *Chlorella* used in this study, which modulated the pink hue imparted by *G. gracilis* aqueous extract. Additionally, the GWY sample exhibited partial phase separation, with White *Chlorella* biomass settling at the bottom of the container (Figure S3, Supporting Information), suggesting limited dispersion stability. This observation warrants a more critical interpretation of the color stability data. Colorimetric measurements were taken at a fixed position on the sample surface and therefore characterized the stability of the color at that location; they are not sensitive to a vertical concentration gradient developing within the container. The low ΔE* values recorded for GWY relative to day 1 (<1.17) thus demonstrate that the color of the upper layer did not change appreciably during storage, but do not exclude a redistribution of the microalgal biomass within the product. It should be noted that phase separation was assessed visually and was not quantified; neither the extent nor the rate of sedimentation was determined.

Previous studies have also explored the use of microalgal pigments as natural colorants in yogurt. For instance, phycocyanin has been reported to remain stable over 21 days of storage at concentrations of 4 wt% and 8 wt% [37]. Similarly, fucoxanthin from *Undaria pinnatifida* increased both a* and b* values on the first day of storage, indicating a shift toward redder and yellower tones [38]. In addition, yogurt enriched with *Limnospira platensis* (formerly Spirulina platensis) at 2 wt% exhibited a stable green coloration of yogurt, originating from the presence of chlorophylls, over 15 days of storage [24]. These findings are consistent with the present results, confirming that algae-derived pigments can maintain color stability in dairy matrices, although their final appearance depends on pigment composition and interactions within the system.

The incorporation of White *Chlorella* markedly modified the color of the yogurt. Relative to GY, the GWY sample showed a 94% reduction in a* (from 3.87 to 0.22), a 78% increase in b* (from 5.75 to 10.21), and a slight decrease in L* (from 80.33 to 78.05), all significant at every sampling point (*p* < 0.05). The pink hue imparted by the *G. gracilis* aqueous extract was therefore largely masked and replaced by a pale cream-yellow tone, attributable to the residual pigmentation of the White *Chlorella* biomass. It is worth noting that a* remained positive in all measurements and was in fact higher than that of the control yogurt (−0.96), so the biomass did not impart a green coloration; the residual color was yellow rather than green, which is consistent with the chlorophyll-deficient character of the strain and with the retention of carotenoid pigments, which are not eliminated by the mutation. Despite this shift, the color of GWY was itself stable throughout storage: ΔE* relative to day 1 remained below 1.17, and the difference relative to the control remained clearly perceptible (ΔE* 4.30–5.42; Table S3). The GWY sample did, however, exhibit partial phase separation, with White *Chlorella* biomass settling at the bottom of the container (Figure S3, Supporting Information), indicating limited dispersion stability.

Previous studies have explored the use of microalgal pigments as natural colorants in yogurt. Phycocyanin has been reported to remain stable over 21 days of storage at concentrations of 4 and 8 wt% [37], and fucoxanthin from *Undaria pinnatifida* increased both a* and b* values on the first day of storage, indicating a shift toward redder and yellower tones [38]. In contrast, yogurt enriched with *Limnospira platensis* (formerly *Spirulina platensis*) at 2 wt% developed a stable green coloration originating from chlorophylls over 15 days of storage [24]. The absence of any green hue in the present work, despite an equivalent level of microalgal incorporation, is precisely the advantage sought when replacing conventional *C. vulgaris* with a chlorophyll-deficient strain. Taken together, these findings confirm that algae-derived pigments can maintain color stability in dairy matrices, although their final appearance depends on pigment composition and on interactions within the system.

### 3.3. pH Evolution of Yogurt

The evolution of pH before fermentation and during the 21-day refrigerated storage period is presented in Figure 1.

A decrease in pH was observed following fermentation, consistent with acidification resulting from starter culture activity. During refrigerated storage, pH remained relatively stable. Similar findings have been reported in yogurt systems containing phycocyanin from *Limnospira platensis*, in which limited changes in pH were observed during 15 and 21 days of storage [37,39]. In those studies, phycocyanin supplementation did not significantly affect the growth of *Streptococcus thermophilus* and *Lactobacillus bulgaricus*. In contrast, other studies have reported decreases in pH during refrigerated storage of algae-containing yogurts, which were associated with changes in lactic acid bacterial populations, including *Lactobacillus acidophilus* and *Bifidobacterium animalis* [40,41]. In the present study, microbiological analyses were not performed; therefore, the observed pH behavior cannot be used to infer the viability or growth of the starter cultures during storage. Moreover, acidification was characterized by pH alone, as titratable acidity was not determined. Because the buffering capacity of the milk matrix allows organic acids to accumulate with only limited changes in pH [42,43], the relatively stable pH observed during refrigerated storage does not exclude continued acid production. Further studies combining pH, titratable acidity, and microbiological monitoring would be required to characterize acidification and bacterial dynamics more comprehensively.

### 3.4. Texture of Yogurt

Texture is a critical quality attribute in yogurt, as it strongly influences consumer acceptance and perception. The incorporation of algae into yogurt and its derivatives can influence the gel network structure, potentially leading to modifications in textural properties over storage time [38,43]. In this context, the texture of yogurt samples with added algae was evaluated by measuring firmness, consistency, cohesiveness, and work of cohesion (Table 4), allowing for a comprehensive assessment of the impact of *G. gracilis* extract and White *Chlorella* on the structural and mechanical properties of the yogurt matrix. Two-way repeated-measures ANOVA indicated that formulation was the dominant source of variation for all four texture parameters (*p* < 0.01; partial η^2^ = 0.79–0.99), whereas storage time had no significant main effect on any parameter (*p* > 0.05; Table S6). A significant formulation × storage time interaction was detected only for the cohesion parameter (*p* = 0.030). Nevertheless, pairwise comparisons among storage times within each formulation revealed no significant changes over the 21-day storage period, as indicated by the lowercase letters in Table 4.

Firmness is the main parameter assessed in yogurt texture analysis, representing the force required to compress the sample. From the seventh day onwards, the three products differed significantly from one another, with the control yogurt being the firmest and the GY sample the softest (e.g., 507.4 ± 3.0 and 275.0 ± 12.1 gf, respectively, at day 14). At day 1 the control yogurt showed considerably greater between-batch variability (432.8 ± 72.4 gf), and its firmness was not significantly different from that of either supplemented formulation; only GY and GWY differed from each other at this sampling point. GWY exhibited higher firmness than GY, corresponding to a 30% difference at Day 1. This pattern is consistent with the higher protein and solids content of GWY, although the present design does not allow the independent contribution of White *Chlorella* to yogurt structure to be established. This indicates a matrix rearrangement in the yogurt with the inclusion of the powdered microalga, likely due to its high protein content, which impacts the texture of the final product. No significant changes in firmness were observed over storage for any of the three formulations.

Consistency followed the same pattern as firmness. From day 7 onwards, CY presented the highest values (8800.0 ± 454.1 to 9821.3 ± 195.8 gf·s), GWY intermediate values (7629.3 ± 287.1 to 7897.2 ± 274.9 gf·s) and GY the lowest (6018.8 ± 62.1 to 6479.7 ± 301.4 gf·s), with all three differing significantly from one another. At day 1, GWY (7940.9 ± 189.4 gf·s) was significantly higher than GY (6477.6 ± 143.4 gf·s), while CY (7862.6 ± 2105.3 gf·s) did not differ significantly from either, again reflecting its greater between-batch variability at this time point. None of the formulations showed significant changes in consistency during the 21 days of storage. The lower consistency of GY relative to CY and GWY may indicate a greater tendency towards syneresis, associated with the water introduced with the aqueous extract.

Cohesiveness, which reflects the strength of internal bonds within the yogurt matrix, showed a similar hierarchy. The GY sample presented the lowest values throughout storage (203.9 ± 10.9 to 229.8 ± 6.5 gf), suggesting a weakening of the gel network associated with incorporation of the aqueous extract, while GWY (305.1 ± 2.6 to 320.7 ± 10.9 gf) and CY (303.9 ± 93.9 to 376.9 ± 22.0 gf) were consistently higher. At days 1 and 7, GWY and CY did not differ significantly from one another. From day 14 onwards CY was significantly the highest of the three. All formulations maintained stable cohesiveness values over the storage period (*p* > 0.05). GWY exhibited higher cohesiveness than GY throughout storage and values closer to those of CY, indicating that the formulation containing both ingredients maintained a stronger gel structure than GY. This difference may be related to the higher protein and solids content of GWY; however, an independent structural effect of White *Chlorella* cannot be established from the present experimental design. This behaviour therefore reflects a difference between both supplemented formulations, GWY and GY, rather than an independently demonstrated effect of White *Chlorella* on the yogurt structure. Similar trends have been reported in the literature. Wang et al. [38] observed that increased acidity over storage reduced yogurt firmness and consistency, whereas Dinçoğlu et al. [41] and Sangian et al. [39] reported enhanced firmness at lower concentrations (0.5 wt%) of *Limnospira platensis* compared with higher levels (0.75–1 wt%). These findings support the importance of optimizing algae concentration to balance functional benefits and textural quality.

The work of cohesion parameter, which measures the energy required to break the internal cohesion of the product, was the only parameter for which a significant formulation × storage time interaction was detected (*p* = 0.030; Table S6). The GWY sample showed the highest values at day 1 (250.1 ± 17.7 gf·s), significantly above both CY (180.9 ± 77.8 gf·s) and GY (181.9 ± 6.5 gf·s), and remained statistically comparable to the control at every subsequent sampling point. GY consistently exhibited the lowest values (161.8 ± 9.4 to 181.9 ± 6.5 gf·s), indicating a weaker and less resilient structure. Although the control yogurt tended to increase over storage (180.9 to 241.7 gf·s), this change was not statistically significant. In conclusion, GWY exhibited work-of-cohesion values comparable to those of CY throughout most of the storage period and higher than those of GY, indicating greater resistance to deformation in the formulation containing both algal ingredients.

### 3.5. Water-Holding Capacity

Water-holding capacity (WHC) reflects the ability of the yogurt gel to retain serum (whey) within its gel structure and is a key indicator of product quality, as whey separation (syneresis) is a natural phenomenon, but it is often perceived negatively by consumers. As a result, yogurts with minimal syneresis are preferred by consumers [44]. Syneresis may occur when the protein network is unable to effectively bind water, which can be influenced by factors such as low pH (< 4.4), inadequate heat treatment, or low total solids content [45].

According to the data presented in Table 5, two-way ANOVA showed that WHC was significantly affected by formulation (*p* = 0.001), by storage time (*p* < 0.001) and by their interaction (*p* = 0.030), with storage time being the factor with the largest effect size (partial η^2^ = 0.90; Table S8). The GY sample presented the lowest WHC at every sampling point, differing significantly from GWY throughout storage (41.38 ± 1.18% versus 45.47 ± 0.60% at day 1, and 45.80 ± 0.40% versus 48.17 ± 0.29% at day 21). This reduction can be attributed to the addition of the aqueous extract, which diluted the total solids and weakened the gel network, reducing its capacity to retain water.

GWY exhibited higher WHC than GY at all sampling points and showed values comparable to, or higher than, those of CY. This pattern may be related to the higher solids content of GWY and to the water-binding properties of proteins and polysaccharides present in the microalgal biomass. However, because no White *Chlorella*-only formulation was included, the independent contribution of the biomass to WHC cannot be distinguished from its effect within the combined GWY formulation. WHC increased significantly during storage in all three formulations, from 44.37 to 46.94% in CY, from 41.38 to 45.80% in GY and from 45.47 to 48.17% in GWY. The significant interaction reflects the different timing of this increase: CY and GY rose significantly by day 14, whereas GWY only differed from its initial value at day 21, indicating that the increase in water-holding capacity followed a more gradual course in GWY than in the other two formulations. These results agree with those reported by Dinçoğlu et al. [41], who observed an increase in WHC with higher concentrations of *S. platensis*, likely due to the water-binding capacity of microalgal proteins and polysaccharides. Conversely, other studies have reported a decrease in WHC during storage, associated with acidification and subsequent weakening of the protein network [24]. In the present study, the stable pH observed throughout storage (Figure 1) is consistent with the progressive increase in WHC rather than with its deterioration.

### 3.6. Total Phenolic Compound Content and Antioxidant Activity

Algae have been gaining increasing attention as a promising natural source of bioactive compounds, and their potential applications in food have been extensively studied [46,47]. In this study, the total phenolic content (TPC) and antioxidant activity of the yogurts after fermentation were evaluated (Table 6). No significant differences (*p* > 0.05) were observed among the samples for TPC, with values ranging from 12.4 to 14.3 mg GAE/100 g. Antioxidant activity showed slight variation among treatments, ranging from 13.2% to 14.5% ABTS radical scavenging activity. The control yogurt presented the highest value (14.5%), differing significantly from the other treatments, while the samples with 13.2% and 13.3% did not differ significantly from each other.

These values should be interpreted with caution. The Folin–Ciocalteu assay is not specific to phenolic compounds: it measures the overall reducing capacity of the sample, and any species able to reduce the phosphomolybdic–phosphotungstic reagent under alkaline conditions contributes to the response [48,49]. In a dairy matrix this is a substantial limitation, since milk proteins react appreciably with the reagent through their tyrosine, tryptophan and cysteine residues, and free amino acids, peptides released during fermentation, and reducing sugars all interfere in the same way [49,50]. Algal matrices add further potential interferents, including ascorbate, thiols and pigments. The comparatively high apparent phenolic content measured in the control yogurt, which contained no algal ingredient, is consistent with a substantial contribution from these non-phenolic reducing species. The values reported here are therefore best regarded as total reducing capacity expressed as gallic acid equivalents, rather than as an absolute quantification of phenolic compounds, and comparisons among formulations are meaningful only in relative terms. Unequivocal identification and quantification of individual phenolic compounds would require chromatographic separation coupled to mass spectrometry, which was beyond the scope of the present study.

No significant differences in either total phenolic content or antioxidant activity were detected between the two supplemented formulations, and the control yogurt showed the highest antioxidant activity of the three. This suggests that the quantity of phenolic compounds introduced with the algal ingredients was insufficient to raise the radical scavenging capacity of the matrix above that already conferred by its intrinsic constituents. Additionally, the use of White *Chlorella*, characterized by reduced chlorophyll content, may further explain the limited antioxidant response, as chlorophyll and its derivatives are known to contribute to antioxidant mechanisms such as free radical scavenging and metal chelation.

Previous studies have shown that the addition of 1% of L. platensis in yogurt can lead to a decrease in antioxidant activity over storage, with DPPH values declining from 77% to 56% after 28 days [41,51]. This reduction has been attributed to C-phycocyanin degradation, potentially caused by the proteolytic activity of lactic acid bacteria such as *L. paracasei* [52]. In addition, the measured antioxidant activity may be influenced by several factors, including the chemical form, stability, and interactions of phenolic compounds, as well as the analytical method employed [53]. These considerations may help explain why the antioxidant activity of the supplemented yogurts was not higher than that of the control in the present study, despite the addition of algal ingredients. It should be noted that sensory evaluation, consumer testing, and microbiological monitoring throughout refrigerated storage were not performed in the present study. Therefore, no conclusions can be drawn regarding sensory acceptability, microbiological stability, or shelf life. These aspects should be addressed in future studies before the practical applicability of the proposed formulations can be established.

## 4. Conclusions

This study demonstrates the technical feasibility of producing yogurt formulations containing *G. gracilis* aqueous extract alone or in combination with White *Chlorella*. The GY formulation exhibited a stable pink coloration throughout fermentation and 21 days of refrigerated storage. Compared with CY, however, GY showed lower firmness, consistency, cohesiveness, and water-holding capacity. Because the control did not contain an equivalent volume of added water, these differences cannot be attributed exclusively to the *G. gracilis* extract and are likely influenced, at least in part, by dilution of the milk solids.

GWY exhibited a different nutritional and physicochemical profile from GY. In particular, GWY contained 32% more protein than GY and showed higher firmness, consistency, cohesiveness, and water-holding capacity, with several parameters approaching or reaching values observed for the control yogurt. These findings show that the formulation containing both algal ingredients had a higher protein content and higher values for several structural parameters than the formulation containing *G. gracilis* extract alone.

No significant differences in total phenolic content were detected among formulations, and the antioxidant activity of both supplemented yogurts was lower than that of the control. In addition, partial sedimentation of the microalgal biomass was observed in GWY, indicating that dispersion stability requires further optimization.

In conclusion, the results establish the preliminary formulation of *G. gracilis* aqueous extract and White *Chlorella* in yogurt formulations. Further studies including sensory evaluation and microbiological monitoring during refrigerated storage are required to assess consumer acceptability and microbiological shelf life.

## Figures and Tables

**Figure 1:**
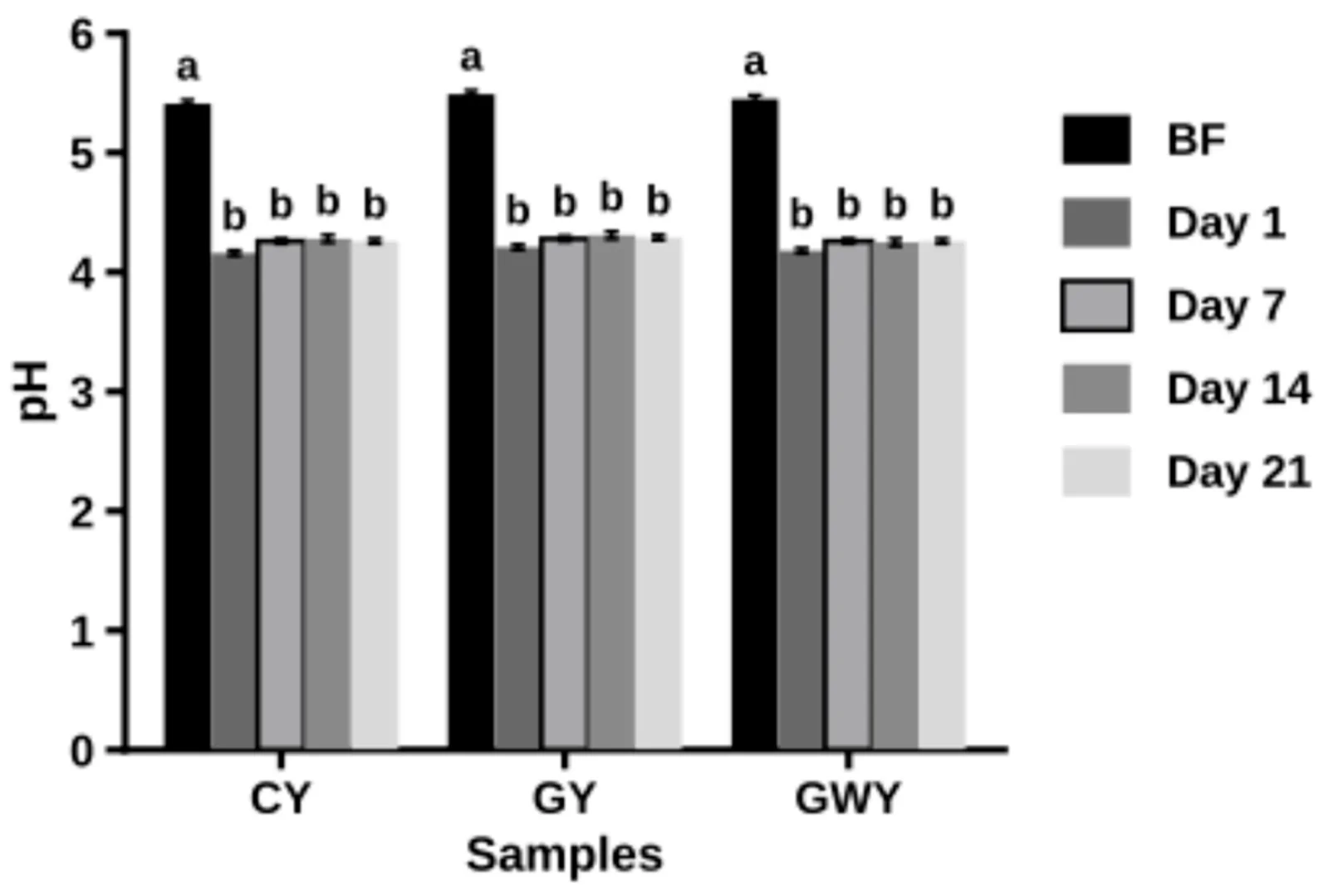
pH values of yogurt samples before fermentation (BF) and during 21 days of refrigerated storage. Values represent the mean ± standard deviation of three independent production batches (*n* = 3). Different lowercase letters indicate significant differences among sampling times within the same formulation. BF: before fermentation; CY: control yogurt; GY: yogurt supplemented with *G. gracilis* aqueous extract; GWY: yogurt supplemented with both *G. gracilis* aqueous extract and White *Chlorella* biomass.

**Table 1:** Proximate composition (protein, carbohydrate, moisture, and ash) and functional properties (TPC and antioxidant activity) of both ingredients used in the yogurt preparation, *G. gracilis* aqueous extract and White *Chlorella* biomass.

	Carbohydrate	Protein	Moisture	Ash	TPC (mgAGE/100 g)	Antioxidant Activity (Inhibition %)
*G. gracilis* aqueous extract (g/100 mL)	0.28 ± 0.01	0.22 ± 0.01	98.2 ± 0.1	0.6 ± 0.05	24.02 ± 2.00	17.47 ± 0.44
White *Chlorella* biomass (g/100 g)	13.40 ± 0.15	45.42 ± 0.13	3.20 ± 0.13	6.0 ± 0.05	-	10.05 ± 0.24

Note: The values represent mean ± standard deviation. Values for *G. gracilis* extract were expressed on a wet basis, while White *Chlorella* biomass values were expressed on a dry basis.

**Table 2:** Proximate composition (g/100 g) of the three yogurts produced by fermentation.

Yogurt Samples	Carbohydrate	Protein	Moisture	Ash
CY	5.05 ± 0.03 ^a^	2.93 ± 0.12 ^a^	88.15 ± 0.03 ^a^	0.80 ± 0.04 ^a^
GY	4.22 ± 0.05 ^b^	2.78 ± 0.10 ^a^	88.40 ± 0.80 ^a^	0.68 ± 0.03 ^b^
GWY	4.05 ± 0.05 ^b^	3.66 ± 0.30 ^b^	87.10 ± 0.20 ^b^	0.71 ± 0.04 ^b^

Note: Values represent the mean ± standard deviation of three independent batches (*n* = 3). Different letters in the same column indicate significant differences among formulations (one-way ANOVA followed by Tukey’s HSD, *p* < 0.05). CY: control yogurt; GY: yogurt supplemented with *G. gracilis* aqueous extract; GWY: yogurt supplemented with both *G. gracilis* aqueous extract and White *Chlorella* biomass. All values are expressed on a wet basis.

**Table 3:** Color parameters (CIELAB color system) for the GY and GWY sample measured over 21 days, and total color difference (ΔE*) relative to day 1 of the same formulation.

Sample	Day	L*	a*	b*	ΔE
GY	1	80.33 ± 0.08 ^aA^	3.87 ± 0.01 ^aA^	5.75 ± 0.03 ^aB^	-
	7	80.60 ± 0.09 ^aA^	3.76 ± 0.00 ^bA^	5.71 ± 0.03 ^aB^	0.31 ± 0.14 ^bB^
	14	79.98 ± 0.28 ^aA^	3.73 ± 0.09 ^abcA^	5.80 ± 0.12 ^aB^	0.42 ± 0.15 ^aA^
	21	80.11 ± 0.03 ^aA^	3.55 ± 0.01 ^cA^	6.08 ± 0.09 ^aB^	0.51 ± 0.06 ^abB^
GWY	1	78.05 ± 0.11 ^bcB^	0.22 ± 0.01 ^bB^	10.21 ± 0.07 ^aA^	-
	7	78.89 ± 0.26 ^abB^	0.25 ± 0.01 ^bB^	9.50 ± 0.01 ^cdA^	1.10 ± 0.20 ^abA^
	14	78.11 ± 0.29 ^acB^	0.55 ± 0.02 ^aB^	9.99 ± 0.12 ^abcA^	0.50 ± 0.14 ^bA^
	21	79.18 ± 0.10 ^aB^	0.23 ± 0.01 ^bB^	9.90 ± 0.10 ^bdA^	1.17 ± 0.01 ^aA^

Note: Values represent the mean ± standard deviation of three independent production batches (*n* = 3); within each batch, four readings were taken and averaged prior to analysis. Data were analyzed by two-way ANOVA with formulation (between-batch factor) and storage time (within-batch factor) and their interaction. The interaction was significant for all parameters (Tables S4 and S5), and simple effects are therefore presented. Different lowercase letters in the same column indicate significant differences among storage times within the same formulation, and different uppercase letters indicate significant differences between formulations at the same storage time (*p* < 0.05). GY: yogurt supplemented with *G. gracilis* aqueous extract; GWY: yogurt supplemented with both *G. gracilis* aqueous extract and White *Chlorella* biomass.

**Table 4:** Texture analysis of yogurt samples for 21 days of storage.

Parameters	Samples	1st day	7th day	14th day	21st day
Firmness (gf)	CY	432.8 ± 72.4 ^aAB^	457.8 ± 22.8 ^aA^	507.4 ± 3.0 ^aA^	508.0 ± 48.1 ^aA^
	GY	292.7 ± 6.4 ^aB^	297.3 ± 16.2 ^aC^	275.0 ± 12.1 ^aC^	287.4 ± 5.4 ^aC^
	GWY	381.4 ± 6.9 ^aA^	384.6 ± 9.6 ^aB^	378.3 ± 2.9 ^aB^	398.5 ± 4.6 ^aB^
Consistency (gf·s)	CY	7862.6 ± 2105.3 ^aAB^	8800.0 ± 454.1 ^aA^	9821.3 ± 195.8 ^aA^	9655.1 ± 943.9 ^aA^
	GY	6477.6 ± 143.4 ^aB^	6479.7 ± 301.4 ^aC^	6244.4 ± 181.3 ^aC^	6018.8 ± 62.1 ^aC^
	GWY	7940.9 ± 189.4 ^aA^	7818.4 ± 181.4 ^aB^	7629.3 ± 287.1 ^aB^	7897.2 ± 274.9 ^aB^
Cohesiveness (gf)	CY	303.9 ± 93.9 ^aAB^	309.8 ± 36.3 ^aA^	368.8 ± 14.8 ^aA^	376.9 ± 22.0 ^aA^
	GY	229.8 ± 6.5 ^aB^	225.2 ± 7.4 ^aB^	203.9 ± 10.9 ^aC^	216.3 ± 5.0 ^aC^
	GWY	320.7 ± 10.9 ^aA^	320.4 ± 23.1 ^aA^	305.1 ± 2.6 ^aB^	318.0 ± 7.6 ^aB^
Work of cohesion (gf·s)	CY	180.9 ± 77.8 ^aAB^	184.4 ± 23.2 ^aB^	222.6 ± 12.4 ^aA^	241.7 ± 5.5 ^aA^
	GY	181.9 ± 6.5 ^aB^	174.7 ± 7.4 ^aB^	161.8 ± 9.4 ^aB^	173.4 ± 2.1 ^aB^
	GWY	250.1 ± 17.7 ^aA^	245.3 ± 6.9 ^aA^	223.0 ± 12.3 ^aA^	234.4 ± 12.3 ^aA^

Note: Values represent the mean ± standard deviation of three independent production batches (*n* = 3), with one determination per batch. Data were analyzed by two-way ANOVA with formulation as a between-batch factor and storage time as a within-batch factor, including their interaction (Table S6); Greenhouse–Geisser-corrected *p*-values were used for the within-batch factor. Different lowercase letters in the same row indicate significant differences among storage times within the same formulation, and different uppercase letters in the same column indicate significant differences among formulations at the same storage time (Tukey’s HSD, *p* < 0.05). CY: control yogurt; GY: yogurt supplemented with *G. gracilis* aqueous extract; GWY: yogurt supplemented with both *G. gracilis* aqueous extract and White *Chlorella* biomass.

**Table 5:** Water holding capacity (%) values for the yogurt samples during 21 days of storage.

Samples	1st day	7th day	14th day	21st day
CY	44.37 ± 0.97 ^bA^	43.60 ± 0.18 ^bB^	45.87 ± 0.51 ^aAB^	46.94 ± 0.92 ^aAB^
GY	41.38 ± 1.18 ^bB^	42.37 ± 0.92 ^bB^	44.89 ± 0.29 ^aB^	45.80 ± 0.40 ^aB^
GWY	45.47 ± 0.60 ^bA^	45.54 ± 0.95 ^bA^	46.42 ± 0.57 ^bA^	48.17 ± 0.29 ^aA^

Note: Values represent the mean ± standard deviation of three independent production batches (*n* = 3), with one determination per batch. Data were analyzed by two-way ANOVA with formulation as a between-batch factor and storage time as a within-batch factor, including their interaction (Tables S7–S9). Different lowercase letters in the same row indicate significant differences among storage times within the same formulation, and different uppercase letters in the same column indicate significant differences among formulations at the same storage time (Tukey’s HSD, *p* < 0.05). CY: control yogurt; GY: yogurt supplemented with *G. gracilis* aqueous extract; GWY: yogurt supplemented with both *G. gracilis* aqueous extract and White *Chlorella* biomass.

**Table 6:** Total phenolic content and antioxidant activity (ABTS radical scavenging) determined for the yogurt samples after fermentation.

Samples	Total Phenolic Content (mgAGE/100 g)	Antioxidant Activity (ABTS Radical Scavenging, %)
CY	13.2 ± 0.5 ^a^	14.5 ± 0.04 ^a^
GY	12.4 ± 0.5 ^a^	13.2 ± 0.5 ^b^
GWY	14.3 ± 0.9 ^a^	13.3 ± 0.5 ^b^

Note: Values represent the mean ± standard deviation of three independent production batches (*n* = 3), with one determination per batch. Data were analyzed by one-way ANOVA followed by Tukey’s HSD test; since these parameters were determined at a single time point, storage time was not included as a factor. Different letters in the same column indicate significant differences among formulations (*p* < 0.05). CY: control yogurt; GY: yogurt supplemented with *G. gracilis* aqueous extract; GWY: yogurt supplemented with both *G. gracilis* aqueous extract and White *Chlorella* biomass.

## Data Availability

The original contributions presented in this study are included in the article/Supplementary Material. Further inquiries can be directed to the corresponding author.

## Supplementary Materials

The following supporting information can be downloaded at: https://www.mdpi.com/article/10.3390/foods15193405/s1, Figure S1: Glucose calibration curve used for the quantification of total carbohydrate content by Dubois method. Absorbance was measured at 490 nm; Figure S2: Gallic acid calibration curve used for the quantification of total phenolic content by the Folin–Ciocalteu method. Absorbance was measured at 765 nm; Figure S3: Photographs of the yogurt samples after 21 days of refrigerated storage at 6 °C; Table S1: Color parameters (CIELAB-color system) for the GY sample during fermentation; Table S2: Color parameters (CIELAB-color system) of GY sample at the end of fermentation (12 h) and of a commercial strawberry-flavoured yogurt; Table S3: Total color difference (ΔE*) of the supplemented yogurts relative to the control yogurt (CY) during 21 days of refrigerated storage at 6 °C; Table S4: Two-way ANOVA for the colour parameters of the supplemented yogurts, with formulation and storage time as factors; Table S5: Verification of the statistical assumptions underlying the analyses reported in Table 3, Tables S3 and S4; Table S6: Two-way ANOVA for the texture parameters of the yogurt samples with formulation and storage time as factors; Table S7: Verification of the statistical assumptions underlying the analyses reported in Table 5 and Table S6; Table S8: Two-way ANOVA for the water-holding capacity of the yogurt samples, with formulation and storage time as factors; Table S9: Verification of the statistical assumptions underlying the analyses reported in Table 5 and Table S8.
