# Supplementary material for: Gracilaria gracilis Aqueous Extract and White Chlorella as Functional Ingredients in Yogurt: Effects on Quality, Nutritional and Physicochemical Properties
Source: Foods. 2026 Sep 24;15(19):3405. doi: 10.3390/foods15193405 (PMC13664106; doi:10.3390/foods15193405)
Supplement: Supplementary file 1 [file foods-15-03405-s001.zip › foods-4519212-supplementary.pdf]

Gracilaria gracilis Aqueous Extract and White *Chlorella* as Functional Ingredients in Yogurt: Effects on Quality, Nutritional and Physicochemical Properties

Gabriela Kovaleski [1], Andreia S. Ferreira [2], Cláudia Nunes [3] and Sónia P. M. Ventura [1,*]

[1] Department of Chemistry, CICECO—Aveiro Institute of Materials, University of Aveiro, Campus
Universitário de Santiago, 3810-193 Aveiro, Portugal; gabrielak@ua.pt
[2] Department of Chemistry, LAQV-REQUIMTE, University of Aveiro, Campus Universitário de Santiago, 3810-193 Aveiro, Portugal; a39493@ua.pt
[3] Department of Materials and Ceramic Engineering, CICECO—Aveiro Institute of Materials, University of Aveiro, Campus Universitário de Santiago, 3810-193 Aveiro, Portugal; claudianunes@ua.pt
* Correspondence: spventura@ua.pt

Figures

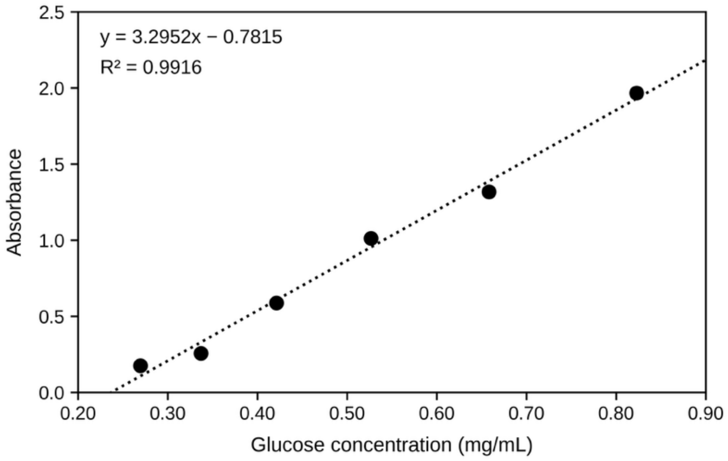

Figure S1. Glucose calibration curve used for the quantification of total carbohydrate content by Dubois method. Absorbance was measured at 490 nm.

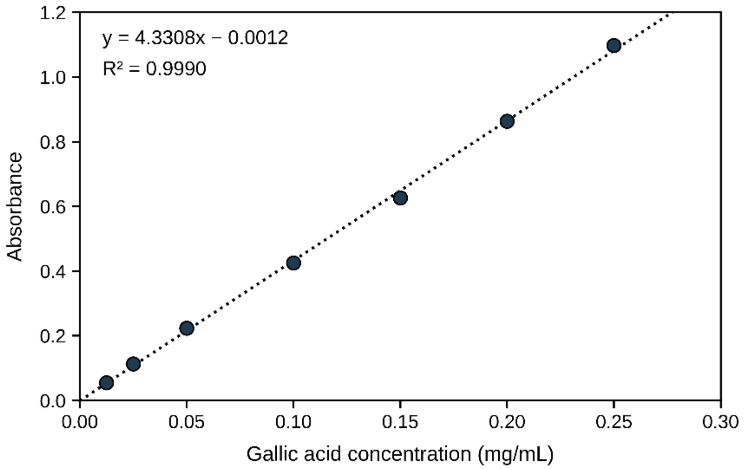

Figure S2. Gallic acid calibration curve used for the quantification of total phenolic content by the Folin–Ciocalteu method. Absorbance was measured at 765 nm.

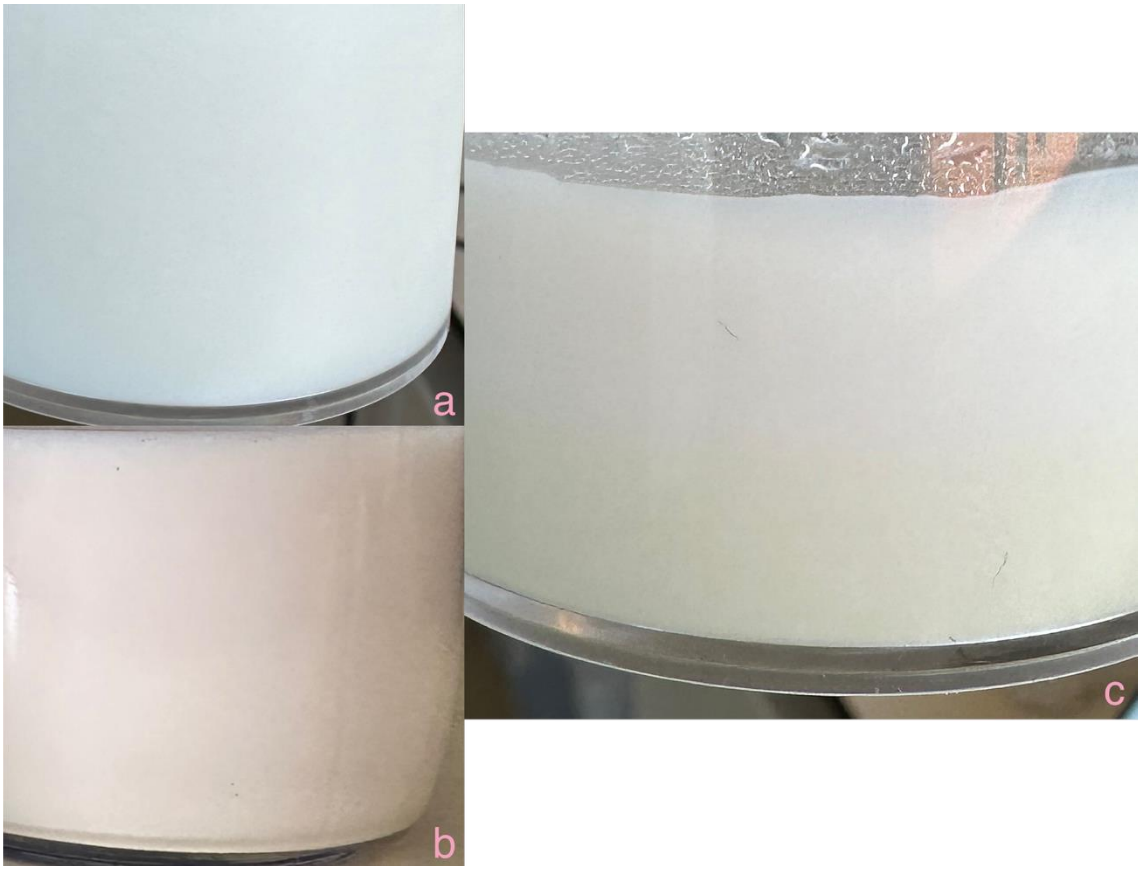

Figure S3. Photographs of the yogurt samples after 21 days of refrigerated storage at 6 °C: (a) CY, control yogurt; (b) GY, yogurt supplemented with *G. gracilis* aqueous extract; (c) GWY, yogurt supplemented with both *G. gracilis* aqueous extract and White Chlorella biomass. The arrow in (c) indicates the partial sedimentation of the microalgal biomass.

Tables

Table S1. Color parameters (CIELAB-color system) for the GY sample during fermentation.

Time (h)	L*	a*	b*	ΔE
**0**	85.19 ± 2.36^{a}	4.82 ± 0.35^{a}	5.38 ± 0.33^{a}	7.43 ± 1.65^{a}
**2**	86.03 ± 2.74^{a}	4.97 ± 0.36^{ab}	5.59 ± 0.23^{ab}	7.33 ± 2.02^{a}
**4**	85.92 ± 2.90^{a}	5.30 ± 0.32^{bc}	5.90 ± 0.29^{bc}	7.56 ± 2.21^{a}
**6**	84.34 ± 2.55^{a}	5.21 ± 0.34^{bc}	6.09 ± 0.30^{c}	8.17 ± 1.91^{a}
**8**	84.45 ± 2.51^{a}	5.18 ± 0.33^{bc}	6.15 ± 0.35^{c}	8.07 ± 1.85^{a}
**10**	85.79 ± 1.10^{a}	5.33 ± 0.18^{c}	5.82 ± 0.49^{c}	7.72 ± 0.78^{a}
**12**	86.33 ± 1.48^{a}	5.17 ± 0.15^{c}	5.82 ± 0.25^{cb}	7.44 ± 1.14^{a}

[1] Values represent the mean ± standard deviation of three independent batches (n = 3). Different lowercase letters in the same column indicate significant differences among fermentation times (one-way ANOVA followed by Tukey's HSD, $p < 0.05$); ΔE* was calculated relative to the blank, CY without added *G. gracilis* extract (L* = 88.98; a* = −1.34; b* = 7.54): GYyogurt supplemented with *G. gracilis* aqueous extract.

Table S2. Color parameters (CIELAB-color system) of GY sample at the end of fermentation (12 h) and of a commercial strawberry-flavoured yogurt.

Samples	L*	a*	b*	ΔE*
Yogurt with *G. gracilis* water extract	86.33 ± 1.48	5.17 ± 0.15	5.82 ± 0.25	2.88± 1.25
Commercial yogurt	83.58 ± 1.26	4.02 ± 0.22	4.62 ± 0.46	-

[1] Values represent the mean ± standard deviation (n = 3). GY was sampled at the end of fermentation (12 h; Table S1). The commercial yogurt contained the added colorants E-100 (curcumin) and E-120 (carmine). ΔE* was calculated for GY relative to the commercial yogurt. GY: yogurt supplemented with *G. gracilis* aqueous extract.

Table S3. Total color difference (ΔE*) of the supplemented yogurts relative to the control yogurt (CY) during 21 days of refrigerated storage at 6 °C.

Formulation	Storage day	ΔE* vs. CY	Perceptibility
GY	1	5.99 ± 0.04 ^{aA}	Clearly perceptible (ΔE* > 3)
GY	7	5.80 ± 0.02 ^{acA}	Clearly perceptible (ΔE* > 3)
GY	14	6.02 ± 0.16 ^{abA}	Clearly perceptible (ΔE* > 3)
GY	21	5.71 ± 0.04 ^{bcA}	Clearly perceptible (ΔE* > 3)
GWY	1	5.42 ± 0.13 ^{aB}	Clearly perceptible (ΔE* > 3)
GWY	7	4.43 ± 0.24 ^{acB}	Clearly perceptible (ΔE* > 3)
GWY	14	5.37 ± 0.24 ^{abB}	Clearly perceptible (ΔE* > 3)
GWY	21	4.30 ± 0.13 ^{bcB}	Clearly perceptible (ΔE* > 3)

[1] Values represent the mean ± standard deviation of three independent production batches (n = 3). ΔE* was calculated against the mean CIELAB coordinates of the control yogurt (L* = 82.93, a* = −0.96, b* = 8.16). Different lowercase letters in the same column indicate significant differences among storage times within

the same formulation, and different uppercase letters indicate significant differences between formulations at the same storage time ($p < 0.05$). Values of ΔE* above 3 correspond to colour differences that are clearly perceptible to an untrained observer. CY: control yogurt; GY: yogurt supplemented with *G. gracilis* aqueous extract; GWY: yogurt supplemented with both *G. gracilis* aqueous extract and White Chlorella biomass.

Table S4. Two-way ANOVA for the colour parameters of the supplemented yogurts, with formulation and storage time as factors.

Parameter	Source of variation	df	F	p-value	p (Greenhouse–Geisser)	Partial η²
L*	Formulation	1, 4	514.11	<0.001		0.992
L*	Storage time	3, 12	20.85	<0.001	0.0369	0.839
L*	Formulation × Storage time	3, 12	14.43	<0.001		0.783
a*	Formulation	1, 4	94853.52	<0.001		1
a*	Storage time	3, 12	50.8	<0.001	0.0793	0.927
a*	Formulation × Storage time	3, 12	50.73	<0.001		0.927
b*	Formulation	1, 4	18432.56	<0.001		1
b*	Storage time	3, 12	27.05	<0.001	0.0479	0.871
b*	Formulation × Storage time	3, 12	20.73	<0.001		0.838
ΔE* vs. day 1	Formulation	1, 4	64.59	0.0013		0.942
ΔE* vs. day 1	Storage time	2, 8	13.19	0.0029	0.0856	0.767
ΔE* vs. day 1	Formulation × Storage time	2, 8	12.73	0.0033		0.761
ΔE* vs. CY	Formulation	1, 4	287.88	<0.001		0.986
ΔE* vs. CY	Storage time	3, 12	36.38	<0.001	0.0103	0.901
ΔE* vs. CY	Formulation × Storage time	3, 12	13.38	<0.001		0.77

[1] Analysis performed on three independent production batches per formulation ($n = 3$), with formulation as a between-batch factor and storage time as a within-batch factor. Greenhouse–Geisser corrected *p*-values are reported for the within-batch factor. Partial η^2 is given as a measure of effect size. ΔE* vs. day 1 was analysed for days 7, 14 and 21 only, since it is zero by definition at day 1. CY: control yogurt.

Table S5. Verification of the statistical assumptions underlying the analyses reported in Table 3 and Tables S3–S4.

Parameter	Shapiro–Wilk W	Shapiro–Wilk p	Levene p	Mauchly W	Mauchly p	Sphericity assumed
**L***	0.943	0.189	0.003	0.174	0.275	Yes
**a***	0.748	<0.001	0.812	0.002	<0.001	No
**b***	0.953	0.321	0.080	0.042	0.043	No
**ΔE* vs. day 1**	0.949	0.405	0.158	0.459	0.211	Yes
**ΔE* vs. CY**	0.958	0.405	<0.001	0.118	0.171	Yes

[1] Normality was assessed by the Shapiro–Wilk test applied to the model residuals, homogeneity of variances by Levene's test, and sphericity of the within-batch factor by Mauchly's test. With three independent batches per formulation these tests have limited statistical power, and residuals were therefore additionally inspected graphically. Where sphericity was not met, Greenhouse–Geisser corrected p-values are reported (Table S4). CY: control yogurt.

Table S6. Two-way ANOVA for the texture parameters of the yogurt samples with formulation and storage time as factors.

Parameter	Source of variation	df	F	p-value	p (Greenhouse–Geisser)	Partial η²
Firmness (gf)	Formulation	2, 6	338.06	<0.001		0.991
	Storage time	3, 18	1.54	0.2383	0.3071	0.204
	Formulation × Storage time	6, 18	1.96	0.1259		0.395
Consistency (gf·s)	Formulation	2, 6	58.63	<0.001		0.951
	Storage time	3, 18	0.77	0.5254	0.4891	0.114
	Formulation × Storage time	6, 18	2.09	0.1057		0.41
Cohesiveness (gf)	Formulation	2, 6	44.56	<0.001		0.937
	Storage time	3, 18	0.75	0.5354	0.5209	0.111
	Formulation × Storage time	6, 18	2.26	0.0845		0.429
Work of cohesion (gf·s)	Formulation	2, 6	11.05	0.0097		0.786
	Storage time	3, 18	0.95	0.4384	0.4914	0.136
	Formulation × Storage time	6, 18	3.08	0.0296		0.507

[1] Values represent the mean ± standard deviation of three independent production batches (n = 3), with one determination per batch. Data were analysed by two-way ANOVA with formulation as a between-batch factor and storage time as a within-batch factor, including their interaction (Table S6); Greenhouse–Geisser corrected *p*-values were used for the within-batch factor. CY: control yogurt; GY: yogurt supplemented with *G. gracilis* aqueous extract; GWY: yogurt supplemented with both *G. gracilis* aqueous extract and White Chlorella biomass.

Table S7. Verification of the statistical assumptions underlying the analyses reported in Table 4 and Table S6.

Parameter	Shapiro–Wilk W	Shapiro–Wilk p	Levene p	Mauchly W	Mauchly p	Sphericity assumed
Firmness (gf)	0.825	<0.001	0.003	0.067	0.003	No
Consistency (gf·s)	0.785	<0.001	0.013	0.052	0.002	No
Cohesiveness (gf)	0.797	<0.001	0.06	0.052	0.002	No
Work of cohesion (gf·s)	0.783	<0.001	0.03	0.072	0.004	No

[1] Normality was assessed by the Shapiro–Wilk test applied to the model residuals, homogeneity of variances by Levene's test, and sphericity of the within-batch factor by Mauchly's test. The departures observed reflect the greater between-batch variability of the control yogurt, particularly at day 1; because the design was fully balanced, ANOVA is robust to deviations of this magnitude (Montgomery, 2019). Where sphericity was not met, Greenhouse–Geisser corrected p-values are reported (Table S6).

Table S8. Two-way ANOVA for the water-holding capacity of the yogurt samples, with formulation and storage time as factors.

Parameter	Source of variation	df	F	p-value	p (Greenhouse–Geisser)	Partial η²
Water-holding capacity (%)	Formulation	2, 6	26.31	0.0011		0.898
	Storage time	3, 18	55.74	<0.001	<0.001	0.903
	Formulation × Storage time	6, 18	3.07	0.0300		0.506

[1] Analysis performed on three independent production batches per formulation (n = 3), with formulation as a between-batch factor and storage time as a within-batch factor. Partial η^2 is given as a measure of effect size. Mauchly's test indicated that the assumption of sphericity was met, and uncorrected p-values are therefore reported for the within-batch factor (Table S9).

Table S9. Verification of the statistical assumptions underlying the analyses reported in Table 5 and Table S8.

Parameter	Shapiro–Wilk W	Shapiro–Wilk p	Levene p	Mauchly W	Mauchly p	Sphericity assumed
Water-holding capacity (%)	0.971	0.461	0.094	0.358	0.232	Yes

[1] Normality was assessed by the Shapiro–Wilk test applied to the model residuals, homogeneity of variances by Levene's test, and sphericity of the within-batch factor by Mauchly's test. All assumptions were met ($p > 0.05$).
